# Optimization of a PVC Membrane for Reference Field Effect Transistors

**DOI:** 10.3390/s90302076

**Published:** 2009-03-19

**Authors:** Chao-Sung Lai, Cheng-En Lue, Chia-Ming Yang, Marek Dawgul, Dorota G. Pijanowska

**Affiliations:** 1 Department of Electronic Engineering in Chang Gung University. / 259 Wen-Hwa 1^st^ Road, Kwei-Shan, Tao-Yuan, R.O.C., Taiwan, 333; 2 Device Section, Department of WAT and Devices, Inotera Memories Inc. / 667, Fuhsing 3^rd^ Road, Hwa-Ya Technology Park, Kwei-Shan, Tao-Yuan, Taiwan; 3 Institute of Biocybernetics and Biomedical Engineering, Polish Academy of Sciences / ul. Trojdena 4, 02-109 Warsaw, Poland.

**Keywords:** PVC membranes, ion-unblocking membranes, REFET, silylating, plasticizers

## Abstract

For the miniaturization of ISFET sensing systems, the concept of a REFET with low ion sensitivity is proposed to replace the conventional reference electrodes through the arrangement of a quasi reference electrode and a differential readout circuit. In this study, an ion-unblocking membrane was used as the top layer of a REFET. To optimize the REFET performance, the influences of the silylating process, different plasticizers, and the composition of the PVC cocktails were investigated. A low sensitivity (10.4 ± 2.2 mV/pH) and high linearity (99.7 ± 0.3 %) in the range from pH 2.2 to pH 11.6 was obtained for the REFET with a 60 wt.% DNP/(DNP + PVC) membrane. To evaluate the long term stability, the drift coefficient was estimated, and for the best REFET, it was −0.74 mV/h. Two criteria for assessing the lifetime of REFETs were used, namely the increase in pH sensitivity to a value higher than 15 mV/pH and the degradation of linearity below 99 %. For the best REFET, it was approximately 15 days.

## 1. Introduction

The ion sensitive field effect transistor (ISFET) was first proposed by P. Bergveld in 1970 [1]. Because the device structure and fabrication process are similar for metal oxide field effect transistors (MOSFETs) and ISFETs, both devices can easily be manufactured by CMOS technology and miniaturized to the micrometer scale [2]. In addition, high bio-compatibility and fast responses have led many researchers to investigate ISFETs as platforms for sensing clinically important species, such as penicillin, urea, glucose, creatinine, etc. [3–7]. Based on these advantages, it has been concluded that ISFETs show high potential for application in “home-care” systems and continuous *in-vivo* monitoring [8].

However, for the purpose of ISFET sensor systems miniaturization, a critical issue for the micro reference electrode (RE) must first be solved [9,10]. To provide a stable reference potential, conventional REs, such as Ag/AgCl or calomel electrodes, filled with an internal electrolyte are used. From the state-of-the-art analysis results, the short lifetime of miniaturized REs with small internal electrolyte volume must still be improved [11,12].

To solve this problem, the concept of a differential system with an ISFET/REFET (reference field effect transistor) pair was first introduced by Matsuo in 1978 [13]. In a REFET, the surface of the sensing membrane for the ISFET was essentially chemically inactivated in order to decrease the pH sensitivity. To replace a conventional RE, an ISFET/REFET pair with a quasi reference electrode (qRE) made of a noble metal, such as Pt or Au, can be used. The output signal of the system, V_out_, obtained in a differential system where V_GS_ of the ISFET (V_ISFET_) and of the REFET (V_REFET_) are both measured versus the common qRE, is as follows:

(1) $$V_{\mathrm{out}}=V_{\mathrm{ISFET}}-V_{\mathrm{REFET}}$$

In this case, the unstable potential of the Pt/solution interface does not influence the output signal, since it is compensated in the differential readout circuit. The concept of an ISFET/REFET differential pair is not only applicable to pH sensing applications, but also to monitoring concentrations of other ions, such as Na^+^ and K^+^ [14], as well as other species, such as creatinine and urea, with the use of chemically and enzymatically modified field effect transistors (ChemFET, EnFET, respectively) [15,16].

Research on REFETs has been based on several approaches, including chemical surface modification, an additional ion-blocking layer, and an ion-unblocking layer deposition. In the first approach, which is based on chemical modification, the surface of the sensing membrane of the ISFET is inactivated by blocking the binding sites. In the case of ion-blocking layer deposition, an extra polymeric layer is cast on the surface of the ISFET. However, the first two methods cause some chemical and electrical problems, as described by Bergveld *et al*. [9]. Their comments imply that an additional ion-unblocking layer with a low conductivity and cation perm-selectivity would be a better solution. A polyvinyl chloride (PVC) membrane has been used to form the ion-unblocking layer on a Si_3_N_4_-ISFET [17]. The pH sensitivity of the REFET decreased to 1.8 mV/pH in the range from pH 2 to pH 9. This indicates that an ion-unblocking layer made by a PVC cocktail might be a good choice for REFET applications, since a reduced sensitivity to hydrogen ions for the REFET and a similar transconductance value for both the ISFET and REFET were obtained. However, the PVC-REFET still has some drawbacks, such as a small operation range, short lifetime, and high drift, which must be improved. Some methods have already been tested [17–19], such as modification of the membrane composition by including additional lipophilic cations, and the use of a buffered poly(2-hydroxyethl methacrylate) (polyHEMA) layer at the interface between the ISFET and the PVC membrane. The polyHEMA layer is frequently used in ChemFETs to decrease the pH sensitivity [20].

To optimize the PVC-REFET in this work, silylating pre-treatment, different plasticizers, and various composition ratios of the PVC cocktail were investigated on standard Si_3_N_4_-ISFETs. To evaluate the sensing properties of REFETs, the sensitivity to hydrogen ions, transconductance compatibility, drift coefficient, and lifetime were studied.

## 2. Experiment

### 2.1. Chemicals

For the silylating process, hexamethyldisilazane (HMDS, Roth, Germany) and toluene (POCh Gliwice, Poland) were used. To form the PVC membrane, high molecular weight polyvinyl chloride (PVC) was purchased from Sigma; the solvent tetrahydrofuran (THF) and three kinds of plasticizers: 2-nitrophenyl octyl ether (*o*-NPOE), bis(2-ethly-hexyl)sebacate (DOS), and dinonylphtalate (DNP), were obtained from Fluka. The salts in this experiment were purchased from POCh Gliwice (Poland). The phosphate buffer solutions of sodium and potassium were prepared in deionized water. The pH value of the buffer solutions were adjusted by adding 0.1 M NaOH and 0.1 M HCl solutions with autoburettes (Mettler-Toledo) and monitored by a combined pH glass electrode.

### 2.2. ISFET fabrication

To maintain electrical isolation between sensors operating in a sensor array, the ISFETs were designed as n-channel devices embedded in p-wells. These ISFETs were fabricated at the Institute of Electron Technology (IET) in Poland. A thermally grown SiO_2_ layer was deposited after RCA cleaning. Afterwards, the Si_3_N_4_ layer, a sensing membrane, was deposited by low pressure chemical vapor deposition (LPCVD). The gate width and length of the transistor channel are 600 μm and 16 μm, respectively. To aid in handmade encapsulation, extended source and drain areas with contact pads located away from the gate area were designed. Finally, all ISFETs were assembled on printed circuit boards (PCB) with a silver paste (TED PELLA, Inc.) and then encapsulated with epoxy resin type adhesive JU-100 (KOKI Company Ltd.) with open windows of 3 × 3 mm^2^.

### 2.3. Optimization of the PVC cocktail for REFETs

**Figure 1.**
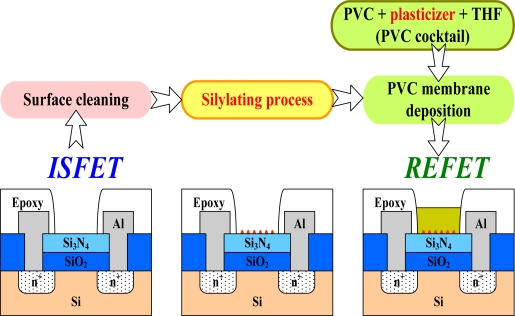
The fabrication process flow for a REFET based on a Si_3_N_4_-ISFET.

In order to decrease the pH sensitivity of the ISFET for REFET application, the PVC membranes were deposited on the open gate windows of ISFETs. The fabrication process flow for the PVC membrane is illustrated in Figure 1. First, the gate insulator surface must be cleaned with deionized water and methanol. Then, for the purpose of chemical grafting and enhancing the adhesion between the PVC membrane and the Si_3_N_4_ layer, a silylating process based on hexamethyldisilazane (HMDS) deposited under different conditions is applied. Then, the PVC cocktail is cast on the Si_3_N_4_ surface of the ISFET with a micro pipette. The solvent from the PVC membrane is evaporated at room temperature overnight.

The procedure for REFET preparation was optimized by tuning the silylating process and PVC membrane composition. The stability and adhesion of the PVC membrane depends mainly on the silylating process; therefore, four silylating processes for the Si_3_N_4_ layer with various HMDS treatments were investigated. The first samples were fabricated with a stock HMDS deposited directly and then dried at room temperature for 15 min, while the second batch of samples was baked at 120°C for 5 min. For the third method, a standard HMDS evaporation process that is used in photolithography was performed at 140°C in a vapor prime oven for 2 min. In the fourth method, HMDS diluted in toluene (ratio = 1:3) was deposited on the surface of the Si_3_N_4_ layer and then dried at room temperature for 15 min.

To prepare the PVC cocktails, three kinds of plasticizers with fixed weight percentage of 70 % were used: *o*-NPOE, DOS, and DNP. In addition, the content of the DNP plasticizer in the PVC cocktail was varied from 50 % to 80 % by weight *vs.* (PVC + DNP). For each batch of the PVC cocktail, six REFET samples were prepared. The total weight of the PVC + DNP was maintained at 200 mg, and all compounds were then dissolved in 3 mL of THF.

### 2.4. Measurement system

To investigate the output signal of the ISFETs and REFETs, a constant drain voltage-constant drain current (CVCC) circuit was adopted to measure the pH sensitivity and long term stability [21,22]. The constant drain-source current (I_DS_) was fixed at 250 μA, and the drain-source voltage (V_DS_) was set at 2.5 V. For the drift coefficient evaluation, all samples were measured in a phosphate buffer (5 mM NaH_2_PO_4_, 0.1 M NaCl) solution of pH 5.7 for 12 h. To evaluate the lifetime of the REFETs, the pH sensitivity and the linearity of the sensor response were checked daily for 1 month. For detailed characterization of the current-voltage curves and transconductances (g_m_) of the ISFETs and REFETs, the drain-source current versus drain-source voltage (I_DS_-V_DS_) and the drain-source current versus the gate-source voltage (I_DS_-V_GS_) characteristics were measured by means of a semiconductor parameter analyzer HP 4156C. To supply a stable reference potential and to obtain the pH characteristics, a conventional Ag/AgCl reference electrode was used as a common grounded electrode in all measurements.

## 3. Results and Discussion

To optimize the sensing properties of the REFET, the silylating process is an important step and was therefore tested first. This step is used to transform the Si_3_N_4_ surface from hydrophilic to hydrophobic and improve the adhesion between the PVC membrane and the ISFET gate material [17]. In this work, the Si_3_N_4_ surfaces of ISFETs were HMDS-silylated under various conditions before the PVC cocktails were cast. All details concerning the process and results are listed in Table 1. The first three silylating processes, based on a stock HMDS deposited under different conditions, failed in the adhesion test. In the last experiment, HMDS was dissolved in toluene to improve the wettability of the Si_3_N_4_ layer by the silylating solution; then, the samples were dried, and the solvent was evaporated at room temperature for 15 min. The best yield and highest linearity of pH response was obtained for the silylating process with a ratio of HMDS:toluene = 1:3.

**Figure 2.**
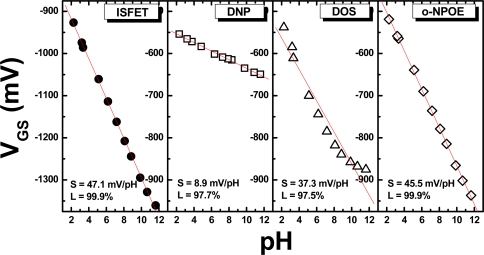
pH sensitivity of (a) a Si_3_N_4_ ISFET without a PVC membrane, and REFETs with membranes containing different plasticizers at 70 wt.% composition: (b) DNP, (c) DOS, and (d) *o*-NPOE.

The second part of the experiment considered the selection of a proper plasticizer for the ion-unblocking membrane. Different plasticizers, including DNP, DOS, and *o*-NPOE, were used. In this experiment, the weight percent of the plasticizers in the PVC cocktail was kept at 70 wt.% for the initial test. The membranes were deposited on the ISFETs treated by the silylating process that produced the best performance, as described earlier, *i.e.* HMDS dissolved in toluene. In Figure 2, the responses of ISFETs and REFETs with different PVC membranes are shown. The lowest sensitivity (8.9 mV/pH) with a linearity of 97.7% was obtained for the REFET with a DNP-based membrane. This PVC membrane decreased the pH sensitivity from 47.1 mV/pH for the ISFET to 8.9 mV for the REFET, as shown in Figures 2a and 2b, while the PVC membranes with DOS and *o*-NPOE plasticizers were still sensitive to hydrogen ions, as shown in Figures 2c and 2d, which excludes them from REFET applications.

**Figure 3.**
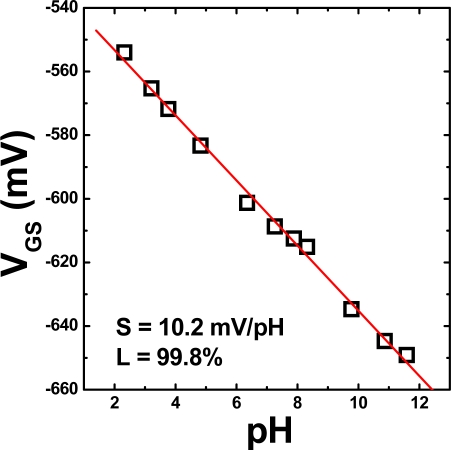
pH response of the REFET with the optimized composition of the DNP solution for the PVC membrane.

In the next stage of this study, the membrane composition was optimized; in particular, the amount of the DNP plasticizer in the membrane was investigated. The weight percent of DNP with respect to total weight of DNP and PVC (i.e. DNP/(DNP + PVC) was adjusted to 50 %, 60 %, 70 %, and 80 %. The sensing properties for REFETs with membranes containing different weight percentages of DNP are listed in Table 2. The plasticizers used in the experiments exhibit different polarity, so that different content of the plasticizers in the membrane results in polarity of the entire membrane and may also influence stability of the membrane. To consider the high accuracy for practical applications of REFETs, apart from the low ion sensitivity, the high linearity of calibration curve should be also taken into account. The lowest pH sensitivity, 10.4 ± 2.2 mV/pH, with the highest linearity was obtained for the REFET with 60 wt. % DNP versus PVC DNP. To verify the previous data, 14 samples were prepared and measured. The pH response of the REFET in the pH range from 2.2 to 11.6 is shown in Figure 3.

**Figure 4.**
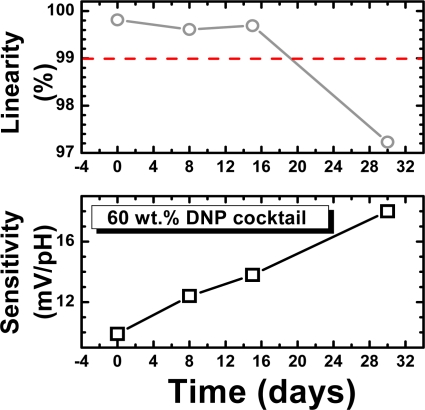
The time-dependent distribution of the sensitivity and linearity of REFETs with PVC membranes fabricated with a 60 wt. % DNP cocktail.

The long term stability and lifetime, which are important parameters for sensor applications, were also investigated. To measure the drift effect of the REFETs, the samples were measured in pH 5.7 buffer solution for 12 hours continuously. The drift coefficient of the REFET with a 60 wt. % DNP membrane was low: −0.74 mV/h. However, after a few days of testing, the reduced pH sensitivity of the REFETs increased, and the linearity degraded, as shown in Figure 4. To evaluate the lifetime of the REFETs, a sensitivity higher than 15 mV/pH and a linearity lower than 99 % were set as the criteria. Based on these criteria, the lifetime of the best REFETs was estimated to be around 15 days.

**Figure 5.**
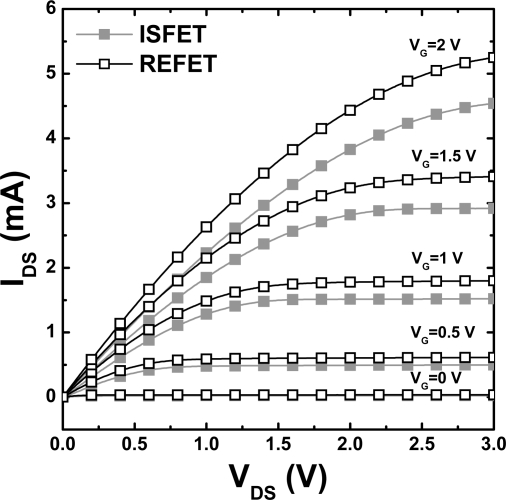
The I_DS_-V_DS_ curves of ISFETs and REFETs with the gate voltage varied from 0 V to 3 V.

In the final stage of this study, the electrical parameters of the optimized DNP/PVC REFETs were tested. The I_DS_-V_DS_ and I_DS_-V_GS_ characteristics of both the ISFET and REFET devices were measured by means of a semiconductor parameter analyzer HP 4156C. In this experiment, the PVC membrane of the REFET was fabricated with a cocktail with the optimized composition, 60 wt% DNP versus DNP + PVC, deposited on top of the HMDS layer obtained with the (1:3) HMDS/toluene solution. I_DS_-V_DS_ curves are similar for both devices (Figure 5). The simplified equation for the I_DS_ of the field effect transistor in the saturation mode is as follows [21]:

(2) $$I_{\mathrm{DS}}=\frac{W\mu_{n}C_{\mathrm{ins}}}{2L}{\left(V_{\mathrm{GS}}-V_{T}\right)}^{2}$$

In Equation (2), W and L are the width and length of the channel, respectively, and μ is the electron mobility. V_GS_ is the voltage bias between the gate and source electrodes, and V_T_ is the threshold voltage. C_ins_ is the capacitance of the Si_3_N_4_/SiO_2_ layer of the ISFET or the PVC/HMDS/Si_3_N_4_/SiO_2_ layer of the REFET. Since the PVC membrane was an ion-unblocking layer that was only permeable for cations [18], the additional series capacitance of the PVC membrane can be ignored. Thus, the C_ins_ of the ISFET and REFET should be the same. Additionally, W, L, and μ are the same for both the ISFETs and REFETs based on the same process and design. Therefore, the higher drain currents of the REFETs depend only on the lower threshold voltage (V_T_) or higher V_GS_ - V_T_.

**Figure 6.**
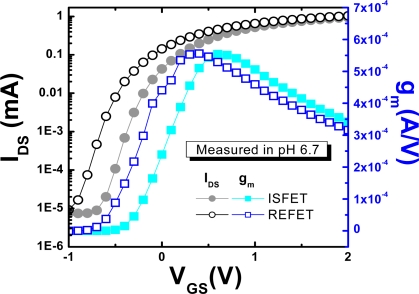
The I_DS_-V_GS_ curves and transconductance (g_m_) of ISFETs and REFETs measured at V_DS_ = 0.5V.

To compare the threshold voltage (V_T_), the on current (I_on_), the off current (I_off_), and the transconductance (g_m_) of ISFETs and REFETs, the I_DS_-V_GS_ characteristics were measured in a pH 6.7 buffer solution (results are shown in Figure 6). The I_on_ and I_off_ for ISFETs and REFETs are almost the same, and the I_on_/I_off_ ratio is about 1.7 × 10^−6^, which is in the normal operation range for FET devices. The threshold voltage for the REFET was smaller than that of the ISFET. The general expression for the threshold voltage for ISFETs and REFETs is as follows [21]:

(3) $$V_{T}=E_{\mathrm{ref}}-\Psi +\chi^{\mathrm{sol}}-\frac{\Phi_{\mathrm{Si}}}{q}-\frac{Q_{\mathrm{OX}}+Q_{\mathrm{SS}}+Q_{B}}{C_{\mathrm{OX}}}+2\phi_{f}$$

In this case, E_ref_ is the potential of the reference electrode, Ψ is the pH-dependent surface potential, and χ^sol^ is the surface dipole potential of the solution. The other terms are contribution of insulator and semiconductor part. All terms in this expression are constant, excluding the pH-dependent surface potential (Ψ). In the case of REFETs, the pH response (shown as a pH-dependent term – Ψ) was suppressed by the additional PVC membrane, which resulted in lower pH sensitivity. Therefore, the smaller value of Ψ and other factors, including the voltage drop across the PVC membrane and the variation of ISFETs’ electrical parameters, resulted in the smaller V_T_ of the REFET (*i.e.* measured in pH 6.7 buffer solution).

In the ISFET/REFET system, the pH response can be obtained with a differential measurement set-up. Therefore, given the common mode rejection ratio (CMRR) in the differential system, the transconductance (g_m_ = dI_DS_ / dV_GS_) of the ISFET and REFET should be the same. As shown in Figure 6, similar transconductances were measured at V_DS_ as 0.5 V and prove that the PVC layer has some electrical conductivity and behaves as an ion-unblocking membrane.

**Figure 7.**
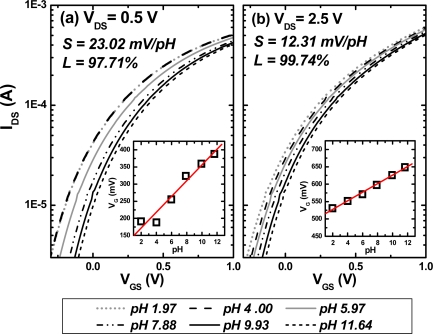
The I_DS_-V_GS_ and sensitivity of REFETs with (a) V_DS_ = 0.5 V and (b) V_DS_ = 2.5 V. Insets show calibration curves corresponding to I_DS_-V_GS_ curves.

In order to find a suitable operation mode for the ISFET/REFET system, V_DS_ was set at 2.5 V and 0.5 V for saturated and unsaturated mode of the field effect transistor operation, respectively. The pH sensitivity was calculated by the corresponding gate-source voltages for different pH buffer solutions at a fixed drain current set to 250 μA. The pH sensitivity and linearity of the calibration curves for the ISFET and REFET are listed in Table 3. The pH sensitivity of the ISFET is almost the same for both values of V_DS_. This phenomenon was also discussed by W. H. Ko in 1982 [23]. The influence of electric field variation resulting from V_DS_ changes around the drain area on the ISFET parameters can be neglected. However, the pH sensitivity and linearity of the REFET depended on the V_DS_. The lowest sensitivity (12.3 mV/pH) and highest linearity (99.7 %) was obtained at V_DS_ = 2.5 V. The I_DS_-V_GS_ curves and pH sensitivities of the REFET are also shown in Figure 7. Therefore, the pH sensitivity of the REFET can be reduced and linearity can be optimized by various V_DS_.

## 4. Conclusions

In this study, the sensing performances of REFETs were optimized by tuning the silylating process, selection of plasticizers, and membrane composition. For optimization, HMDS: toluene = 1:3 was the best silylating mixture for REFETs, resulting in improved adhesion of the PVC membrane to the Si_3_N_4_ surface. The REFET with DNP as a plasticizer had a lower pH sensitivity than REFETs with membranes containing other plasticizers. The lowest sensitivity (10.4 ± 2.2 mV/pH) with high linearity (99.7 ± 0.3%) was found for the REFET with a 60 wt.% DNP membrane. This indicates that the PVC membrane can be used to decrease the pH sensitivity of Si_3_N_4_-ISFETs. The drift coefficient for REFETs with optimized PVC membranes was −0.74 mV/h, and the lifetime was approximately 15 days.

## Figures and Tables

**Table 1.** Silylating process for Si_3_N_4_-ISFETs (RT = room temperature).

**Silylating processes**	**Yield (%)**	**Linearity (%)**
HMDS, RT, 15 min	0	w/o
HMDS, 120°C, 5 min	50	<95.3
Standard HMDS evaporation, 140°C, 2 min	100	<92.3
HMDS:toluene (1:3), RT, 15 min	100	98.6

**Table 2.** Performance of REFETs with PVC membranes with different contents of DNP.

**DNP/PVC + DNP**	**Drift (mV/h)**	**Sensitivity (mV/pH)**	**Linearity (%)**
50 wt. %	−0.70	Unstable	w/o
60 wt. %	−0.74	10.4 ± 2.2	99.7 ± 0.3
70 wt. %	−1.05	9.2 ± 1.2	97.6 ± 1.3
80 wt. %	−0.21	35.0 ± 2.9	97.4 ± 1.1

**Table 3.** The pH sensitivity and linearity of ISFETs and REFETs measured by a HP 4156C with V_DS_ at 0.5 V and 2.5 V.

	**V_DS_ = 0.5 V**		**V_DS_ = 2.5 V**	
	**Sensitivity (mV/pH)**	**Linearity (%)**	**Sensitivity (mV/pH)**	**Linearity (%)**
**ISFET**	46.7	99.9	48.2	99.9
**REFET**(60 wt.%-DNP)	22.9	97.7	12.3	99.7
